# Climate Change and Aging: Implications for Psychiatric Care

**DOI:** 10.1007/s11920-024-01525-0

**Published:** 2024-08-30

**Authors:** Michelle M. Mehta, Anne E. Johnson, Badr Ratnakaran, Ioana Seritan, Andreea L. Seritan

**Affiliations:** 1 White Earth Tribal Behavioral Health, P.O. Box 300, White Earth, MN 56591 USA; 2 Department of Psychiatry, University of Texas Southwestern Medical Center, 5323 Harry Hines Blvd. #9070, Dallas, TX 75930 USA; 3 Department of Psychiatry and Behavioral Medicine, Carilion Clinic-Virginia Tech Carilion School of Medicine, 2017 S. Jefferson St., Roanoke, VA 24014 USA; 4 American Birding Association, Colorado Springs, CO 80934 USA; 5 Department of Psychiatry and Behavioral Sciences, University of California, San Francisco, 675 18th St., San Francisco, CA 94107 USA; 6 UCSF Weill Institute for the Neurosciences, San Francisco, USA

**Keywords:** Climate change, Older adults, Climate disasters, Extreme temperature, Heatwaves, Air pollution

## Abstract

**Purpose of Review:**

We reviewed recent evidence regarding the impact of climate change (specifically, high ambient temperatures, heatwaves, weather-related disasters, and air pollution) on older adults’ mental health. We also summarized evidence regarding other medical problems that can occur in aging adults in connection with climate change, resulting in psychiatric manifestations or influencing psychopharmacological management.

**Recent Findings:**

Older adults can experience anxiety, depressive, and/or posttraumatic stress symptoms, as well as sleep disturbances in the aftermath of climate disasters. Cognitive deficits may occur with exposure to air pollutants, heatwaves, or post-disaster. Individuals with major neurocognitive disorders and/or preexisting psychiatric illness have a higher risk of psychiatric hospitalizations after exposure to high temperatures and air pollution.

**Summary:**

There is a growing body of research regarding psychiatric clinical presentations associated with climate change in older adults. However, there is a paucity of evidence on management strategies. Future research should investigate culturally appropriate, cost-effective psychosocial and pharmacological interventions.

## Introduction

Climate change is a major public health emergency, which disproportionately affects vulnerable populations, including older adults, and amplifies health care inequities [1, 2]. Extreme temperatures (including heatwaves) and weather disasters (e.g., hurricanes, storms, floods, tsunamis, wildfires, and mudslides) have been best studied. Slow-moving disasters such as droughts, sea level rise, increases in water and soil salinity, and crop failures also cause human and property loss and can have long-lasting mental health consequences [1, 2]. Older adults are among the groups most vulnerable to climate change effects [1, 2]. A range of physiological age-related changes, including lower hemoglobin levels, lower cardiac output, reduced glomerular filtration rate, decreased respiratory capacity, reduced sweating, and decreased thirst contribute to this heightened vulnerability [3–5]. Aging adults are more susceptible to dehydration which, in turn, predisposes them to complications such as renal injury, seizures, and hypovolemic shock during heatwaves. Geriatric patients may also have chronic respiratory, cardiovascular, or renal disease, which further increase their morbidity and mortality due to extreme temperatures and natural disasters. Moreover, sensorimotor deficits, cognitive impairments, or linguistic isolation can impair their ability to escape to safety [6]. Additionally, mounting evidence links air pollution to cognitive decline, neurodegenerative diseases, and late-life depression [7–10].

Multiple social determinants of health converge with the age-related vulnerabilities noted above to amplify the impacts of climate change worldwide [1, 2]. Women, Indigenous people, racial/ethnic minorities and other minoritized populations, economically disadvantaged groups, outdoor workers, people living in low-lying areas prone to sea level rise and flooding, and those residing in urban areas with limited green spaces are at higher risk [2, 11–16]. People with severe mental illness (SMI) are also more likely than those without mental illness to be unemployed, insecurely housed, socially excluded, live in poverty, or be incarcerated, which further increases their risk of adverse events with natural disasters or when facing extreme temperatures [17, 18]. The intersection of social and ecological determinants of health poses additional challenges for prevention, management, and designing effective community emergency preparedness plans.

There have been several recent reviews focusing on the mental health impacts of climate change for the aging population [4, 6, 19]. Most of the previous work has either focused on specific climate disasters, such as hurricanes or wildfires [20–24] or extreme heat [25, 26] and the associated psychiatric manifestations. Here we propose a novel framework, based on settings of care (emergency, medical, inpatient psychiatric, and outpatient). We review extant evidence, especially that published in the last decade, and provide management recommendations for each setting. Knowing that older adults are even more vulnerable to adverse outcomes during transitions of care, we aim to discuss a continuum of care, ranging from acute presentations in the aftermath of natural disasters, to outpatient clinical scenarios.

### Case Vignette

Ms. C is a 73-year-old woman brought to an emergency department after a psychiatry resident volunteering at a shelter for hurricane evacuees recommended evaluation for altered mental status and dehydration. Ms. C had refused food and water offered to her and was hesitant to leave the bus that had transported her 500 miles from a temporary shelter to a neighboring Gulf coast state. Ms. C appeared confused, smelled of urine, and had an unsteady gait.

In the emergency room, Ms. C was minimally oriented and had no personal belongings or identification documents with her. She was observed talking to herself and crying, at one point saying that she saw a dead body. She appeared distracted, frightened, and paranoid when approached by hospital staff and startled easily. She eventually allowed a nurse to check her vital signs and draw blood. Findings indicated markers of dehydration as well as a urinary tract infection (UTI). She later accepted IV fluids and antibiotics. When offered food, she appeared suspicious but then ate and drank a small amount before sleeping briefly. After one dose of antipsychotic medication, her mental status improved slightly. However, during psychiatry consultation, she was noted to have ongoing paranoid delusions and disorganized thought process and was admitted involuntarily to the hospital’s geriatric psychiatry service.

During the first week of her hospitalization, Ms. C slept poorly, frequently waking up her roommate by yelling out during nightmares. During the day she isolated from peers and was irritable, mumbled to herself, needed reassurance from staff regarding her safety, and repeatedly requested to be discharged. After a few days of antipsychotic medication treatment and improved sleep, she began to interact with select peers and sought out conversation with the staff. She provided details of her medical history, significant for diabetes, hypertension, hypothyroidism, asthma, sleep apnea, overactive bladder, and depression. She had been taking metformin, lisinopril, levothyroxine, albuterol, oxybutynin, and venlafaxine. She eventually shared her brother’s name for collateral information. She did not want to talk about her experience during the hurricane and avoided watching news on television. Her Mini-Mental State Examination (MMSE) score was 21/30, missing points for orientation (3), recall (3), repetition (1), and serial 7s (2).

When the team reached her brother, he was relieved to hear that Ms. C was alive. In their last phone conversation, he had implored her to evacuate before the hurricane, but Ms. C did not want to leave her home or her dog; finances were tight after home repairs due to prior flooding, and she was nervous driving long distances with her pet. He explained that Ms. C was recently widowed. She was a retired middle school teacher and enjoyed gardening and walking her dog. She had been less active since a knee replacement surgery six months prior. Ms. C’s brother called her regularly as he was worried about her seeming lonely and forgetful at times. Their conversations that summer focused on her worries about her health, the heat, and mosquitoes as well as the tense relationship with her only child. He was not aware of any prior psychiatric treatment, and there was no family history of mood or psychotic disorders.

Later in her hospitalization, Ms. C shared some details of the trauma she experienced during the hurricane, including the drowning of her dog when the first floor of her house flooded. A taper in antipsychotic dose was attempted but paranoid ideation returned; she was stabilized on a combination of antipsychotic and antidepressant medication and at the time of hospital discharge had improved mood, sleep, and oral intake, and no evidence of psychosis.

This vignette illustrates several aspects of older adults’ experiences related to climate change: potential exacerbation of underlying medical problems and anxiety with increased temperatures; natural disasters causing severe trauma and personal or property loss, including loss of beloved pets; the impact of successive disasters; displacement to temporary shelters, with limited access to basic necessities or medications; physical aspects, such as dehydration, UTI, and being prescribed medications which increase the risk of heat-related illness; and psychiatric sequelae of natural disasters, such as anxiety, insomnia, depression, psychosis, and posttraumatic stress symptoms. Additionally, older adults are more vulnerable due to cognitive impairment and social isolation. Each of these aspects will be discussed in detail in the following sections.

## Acute and Medical Care Settings

In this section, we will discuss climate change-related clinical presentations that may be encountered in older adults at post-disaster triage points, in the community, temporary shelters, emergency departments (EDs), and in general hospital settings.

### Post-Disaster Triage Points and Shelters

Natural disasters can cause acute direct and indirect health consequences. Disaster exposure can lead to adjustment disorders, anxiety, acute or posttraumatic stress symptoms, depression, sleep disturbances, substance misuse, and suicidality [6, 19, 24]. The prevalence of post-disaster PTSD among older adults can reach 50% [20]. Successive disasters in the same area can retraumatize survivors and exacerbate posttraumatic stress symptoms. Immigrants, refugees, veterans, and others with prior history of trauma may be particularly vulnerable. In areas affected by Hurricane Sandy in the U.S., 14% of older adults reported clinically significant depressive symptoms, and 5% endorsed suicidal ideation 2 years post-disaster [22]. Displaced survivors, especially when separated from their families, may feel overwhelmed, sad, angry, frightened, or mistrustful in a suddenly changed, unstable environment (as was the case for Ms. C, in the vignette above) [11, 24]. Additional psychiatric and cognitive manifestations related to climate change are discussed in the “Outpatient Settings” section.

Destruction of infrastructure during natural disasters impedes access to life-saving health care services such as hemodialysis, while power outages compromise the use of durable medical equipment like portable oxygen supply [27]. There is also an increased risk of death through carbon monoxide poisoning due to unconventional heating devices. Nursing home residents are at even higher risk during natural disasters compared to community-dwelling older adults [28]. Moreover, evacuation of residents from long-term care facilities requires arranging the transport and transfer of medical equipment, medications, and records, all of which can be disrupted by climate emergencies, amplifying the risk of post-evacuation mortality [29]. Older adults can also face the additional challenges of post-disaster displacement and migration, resulting in disruption of established social connections, limited access to basic needs, language barriers, or complex immigration processes for those who relocate to other countries [11].

### Medical Emergencies and Medical Settings

A study conducted in areas affected by Hurricane Sandy revealed a significantly higher utilization of local EDs in the 3 weeks after the hurricane relative to pre-landfall, especially for people ≥85 years old [23]. The main ED visit reasons were need for hemodialysis, electrolyte imbalances, and prescription refills [23]. Older adults can also present to EDs for various physical symptoms caused or exacerbated by temperature changes or air pollution [3, 28, 30–33]. Heatwaves have been linked to higher rates of hospitalizations among adults ≥ 65 years old [31]. Fluid and electrolyte imbalances, renal failure, urinary tract infections, sepsis, and heat stroke were common concerns [32]. Furthermore, many psychotropic medications can increase the risk of heat stroke [5, 6]. Antipsychotic and anticholinergic agents were associated with a significantly higher risk of death for older adults during heatwaves [31]. Aging individuals who were prescribed antipsychotics had higher odds of being hospitalized during summer months, even in the absence of heatwaves, particularly if they had chronic kidney disease or diabetes [31]. A U.K. study also revealed increased death rates associated with elevated temperatures among individuals with dementia, psychosis, or substance use disorders [34]. However, younger people (< 65 years) were found to be at higher risk compared to those above age 65 in this study.

Climate change has resulted in an expanding range of vector-borne (e.g., Lyme disease, West Nile virus) or non-vector-borne infectious diseases such as coccidioidomycosis, which can present with delirium or other neuropsychiatric manifestations [35–38]. A recent scoping review also revealed a higher risk of stroke with weather extremes and greater temperature variability, although these findings were not age-specific [35].

Table 1 summarizes climate change effects on various organ systems and considerations when prescribing psychotropic medications to older adults [3, 4, 23, 30, 31, 33–49].

**Table 1:** Climate change effects on various organ systems and considerations when prescribing psychotropic medications

**Organ system**^a^	**Climate change-related factors**	**Climate change-related effects in older adults**	**Considerations when prescribing psychotropic medications**
Cardiovascular [3, 4, 30, 31, 39, 40]	Air pollution Extreme temperatures Heatwaves	Increased risk for myocardial infarction, heart failure, cardiac arrhythmias, hypovolemic shock (with dehydration) -Hypertensive emergencies post-natural disasters Increased risk of heat-related illness, including heat stroke, during heatwaves Accelerated coronary atherosclerosis due to air pollution	Educate patients and caregivers regarding increased risk of heats stroke when prescribing psychotropic medications that can cause dehydration or impair thermoregulatory mechanisms including antipsychotics, beta-blockers, SSRIs, and agents with anticholinergic properties (e.g., first-generation antipsychotics, clozapine, olanzapine, tricyclic antidepressants, benztropine, trihexyphenidyl) Counsel patients to maintain adequate hydration and avoid prolonged exposure to heat Be cautious when prescribing psychotropic medications that can cause tachycardia or prolong QTc interval (e.g., haloperidol, chlorpromazine, pimozide, pimavanserin, quetiapine, ziprasidone, donepezil, methadone, stimulants, citalopram, venlafaxine, and tricyclic antidepressants)
Neuropsychiatric [30, 35–38, 41–44]	Air pollution Extreme temperatures Heatwaves	Significantly increased risk of cognitive deficits associated with exposure to PM_2.5_, black carbon, and NO_2_ Increased risk of seizures with dehydration, especially in patients with epilepsy Exacerbation of migraine and multiple sclerosis symptoms with heatwaves Increased risk of ischemic stroke with larger diurnal temperature variation, and of both ischemic stroke and intracerebral hemorrhage with lower temperatures Expanding geographic range of infectious diseases with neuropsychiatric symptoms (e.g., Lyme disease, West Nile virus, coccidioidomycosis)	Be cautious when prescribing medications that can worsen cognition or contribute to delirium (e.g., benzodiazepines, opioids, agents with anticholinergic properties) Be cautious when prescribing agents that can lower seizure threshold (e.g., bupropion, clozapine) or cause hyponatremia (e.g., SSRIs, SNRIs, clomipramine, carbamazepine, oxcarbazepine), as electrolyte imbalances can increase risk of seizures Be cautious when prescribing SSRIs or SNRIs, as they can increase bleeding risk Be cautious when prescribing antipsychotics, as they can worsen motor symptoms of Parkinson’s disease and are associated with increased risk of cerebrovascular events and mortality in older adults with major neurocognitive disorders
Pulmonary [4, 36, 45]	Air pollution Extreme temperatures Heatwaves Longer pollen (allergen) seasons Mold exposure after floodings	Worsening of asthma, chronic obstructive pulmonary disease, and other respiratory diseases Increased risk of viral respiratory illness and/or viral pandemics due to weather-related human behavior changes (e.g., limited outdoor activity)	Be cautious when prescribing medications that can cause respiratory depression (e.g., benzodiazepines, gabapentin, pregabalin, opioids)
Renal [4, 23, 33, 46]	Extreme temperatures (especially higher ambient temperatures) Heatwaves Hurricanes and floods	Increased risk of kidney stones and urinary tract infections due to dehydration Heat-induced inflammatory kidney injury, rhabdomyolysis from heat stroke, and chronic kidney disease	Counsel patients to maintain adequate hydration and avoid prolonged exposure to heat Be cautious when prescribing lithium, as dehydration can increase lithium levels and cause toxicity; long-term use of lithium can also cause interstitial nephritis and increase risk of kidney failure Adjust medication doses as necessary in patients with renal impairment
Gastrointestinal [4, 36, 47]	Hurricanes and floods	Increased risk for hepatitis and waterborne gastrointestinal infections Intestinal microbiome changes	Be cautious when prescribing psychotropic medications that can be hepatotoxic (e.g., valproic acid, carbamazepine, chlorpromazine, clozapine) Adjust medication doses as necessary in patients with hepatic impairment
Skin [5, 36, 48, 49]	Air pollution Increased UV exposure due to ozone layer depletion Extreme temperatures	Increase in atopic dermatitis and eczema Increased incidence of melanoma and non-melanoma skin cancers (basal cell carcinoma, squamous cell carcinoma) Frostbite	Advise patients to wear sunblock, hats and loose-fitting lightweight clothing for UV protection and limit time spent outdoors during heatwaves and summer days or in extremely cold weather Be cautious when prescribing agents that impair vasodilation of skin capillaries, such as beta-blockers and sympathomimetics

*NO*_*2*_ nitrogen dioxide, *PM*_*2.5*_ particulate matter with diameter smaller than 2.5 µm, *SNRIs* serotonin-norepinephrine reuptake inhibitors, *SSRIs* selective serotonin reuptake inhibitors, *UV* ultraviolet

^a^Climate change-related psychiatric manifestations are discussed in detail in text and Table 3

Suggested laboratory workup for older adults presenting for care during heatwaves or after natural disasters includes complete blood count, electrolytes, renal and liver function tests, urinalysis, and electrocardiogram, followed by chest radiograph and urine and blood cultures, if there is a clinical suspicion of infection or a presentation consistent with delirium. Screening for coronavirus disease 2019 (COVID-19) and other community-acquired infections should be considered for individuals living in high-density settings, such as shelters, nursing homes, or prisons. Additional tests such as lumbar puncture can be performed if encephalitis is suspected. Neuroimaging studies like head computerized tomography can rule out acute vascular events or subdural hematoma secondary to trauma or falls during the disaster.

### Post-disaster Psychiatric Emergencies

Our vignette discussed an older woman with no prior psychiatric history who presented confused and paranoid after surviving a hurricane and witnessing destruction and death. Morbidity and mortality reports published in the aftermath of disasters, such as Hurricane Katrina, captured the presence of psychosis and confusion, although the exact prevalence and age-stratified data were not available [50]. Older adults, particularly those with preexisting cognitive deficits, are at risk of delirium due to dehydration, electrolyte imbalances, infections, or abrupt discontinuation of alcohol or benzodiazepines. People who survive disasters may also develop new-onset psychotic symptoms [24, 51]. Direct exposure and having a friend or family member who died or was injured in the disaster increase the risk for psychosis [51]. The Diagnostic and Statistical Manual of Mental Disorders (DSM-5) recognizes brief psychotic disorder with marked stressors (brief reactive psychosis) [52], which has also been reported in uninfected older adults during the COVID-19 pandemic [53].

There is also a concern for increased suicide risk among older adults in the aftermath of disasters. Older adults have high rates of suicide worldwide; depression, bereavement, and loss of physical health or independence are important risk factors [54]. As consequences of climate change-related events can be intertwined with these factors, it is imperative to screen patients for suicidal ideation and behaviors and institute safety protocols. Additionally, older women are at risk for gender-based violence during disasters and displacement [55, 56]. This should be kept in mind when evaluating older adults in post-disaster settings, especially women.

### Management

Aging adults presenting to temporary shelters or EDs in the aftermath of natural disasters should be promptly assessed, and high priority given to those with immediate medical or mental health needs. Various triage systems exist worldwide to support disaster victims. Goldmann et al. [57] developed a behavioral health assessment module comprising 26 questions focused on mental health and substance use that can be administered in person, by phone, or online.

Providing a sense of safety and security is the first step in supporting disaster survivors. Psychological First Aid (PFA) is an evidence-based approach designed to reduce stress and help recovery from trauma by improving adaptive functioning and coping [58]. PFA can be conducted in multiple sessions and has been beneficial for older adults [59], although not specifically with weather disasters. PFA core components include providing safety, comfort, and stabilization; gathering information regarding current needs and concerns; offering practical assistance; connecting with social support and other services; and education on coping with disasters [58].

## Psychiatric Admissions and the Inpatient Setting

There is a large international body of knowledge underscoring the association of climate change-related factors with increased utilization of mental health services – among these, psychiatric hospitalizations. More frequent ED visits and psychiatric admissions associated with extreme temperatures occur across a wide range of mental health conditions including psychotic disorders [16, 60–63], mood disorders [26, 60, 62–64], substance use disorders [60, 61], anxiety disorders [60], autism spectrum disorders [60], and major neurocognitive disorders (NCDs) such as Alzheimer’s disease (AD) [13, 65, 66]. Higher levels of air pollutants have also been associated with increased hospitalization rates for people with schizophrenia, depression, bipolar disorder, and major NCDs [26, 63, 65]. Pollutants studied include particulate matter less than 2.5 μm in diameter (PM_2.5_), particulate matter less than 10 μm in diameter (PM_10_), nitrogen dioxide (NO_2_), ozone (O_3_), and sulphur dioxide (SO_2_), among others. Consistent with prior reviews [2, 4, 6], older adults, women, and socioeconomically vulnerable groups often face disproportionate morbidity. However, a few studies found males or younger or middle-aged adults [34, 61, 67] to be at higher risk compared to women and elderly, respectively, and postulated these groups might have higher occupational exposure to outdoor weather conditions.

When examining the impact of heatwaves, studies have focused on the association of mental health emergencies with heatwave occurrence, duration, intensity, or lag days (the period immediately following a heatwave). Presence of major NCDs increases the risk of ED visits and hospitalizations at baseline, with most common reasons being falls, weakness, and altered mental status [68]. People with major NCDs are even more vulnerable to extreme temperatures than those without major cognitive impairments and therefore more likely to be hospitalized [13, 16, 35, 65]. Gong and colleagues found that the risk of dementia-related hospitalization increased by 4.5% for every 1 °C above 17 °C, and the risk was more than double for individuals ≥ 75 years old, compared to the 16–74 age group [13]. Culqui et al. [65] uncovered a significant association between AD-related emergency admissions and maximum daily temperature during heatwaves in Madrid. Neighborhood green spaces are protective, by providing a cooling effect and absorbing carbon dioxide from the atmosphere. Xu and colleagues showed that older adults who resided in places with lower green space surface had a higher risk of being admitted for AD-related reasons during heatwaves compared to those living in neighborhoods with more green space [69].

Mental health hospitalizations (all-cause or for causes other than major NCDs) have also been associated with high ambient temperatures and air pollution. Chan and colleagues examined data from Hong Kong over a decade and found that adults > 75 years old had a higher risk of psychiatric admissions with increased temperature compared to younger counterparts [26]. A study focusing on the U.S. Medicare population found a significantly higher risk of acute psychiatric hospitalization for depression, bipolar disorder, and schizophrenia with increased ambient temperature and exposure to air pollutants (though these associations were present only in the cold season) [63]. In contrast, Wei et al. uncovered a *lower* risk of dementia-related hospitalizations in years with higher-than-average temperatures (both in summer and winter months), although there was a *higher* risk of admissions due to dementia in geographic areas with higher temperature variability [66].

Table 2 summarizes recent studies reporting on the association of ambient temperature and/or air pollution with admissions for psychiatric illness or major NCD among older adults [13, 16, 26, 60–66, 69].

**Table 2:** Studies showing association of ambient temperature and/or air pollution with hospitalizations for psychiatric illness or major neurocognitive disorders among older adults

**Study author, year**	**Location, study period**	**Study sample**	**What was studied?**	**Key findings**	**Age-related findings**
Chan et al. 2018 [26]	Hong Kong, 2002–2011	44,600 mental health admissions	Effects of ambient temperature on mental health hospitalizations	Mental health hospitalization cumulative RR for all age groups at 28 ˚C, compared to 19.4 ˚C was 1.09.	Adults ≥ 75 years old had the highest RR (1.20) with increased ambient temperature.
			Effects of four air pollutants (PM_10_, NO_2_, O_3_, SO_2_) on mental health admission rates	Risk of hospitalization rose with NO_2_ levels > 90 µg/m^3^ (approx. 95th percentile), particularly in older adults.	RR 1.19 for adults 60–74 years old, and 1.49 for those ≥ 75 years old.
Corvetto et al. 2023 [60]	Multiple studies and locations	Not reported	Global systematic review of the impact of climate change on the demand for mental health services	Suicidal behavior and morbidity from mood disorders, anxiety disorders, PTSD, schizophrenia, and substance misuse increased after natural disasters. Mental health-related emergency visits and hospitalizations were significantly higher during hot periods, especially lag^a^ day 5–7.	Increased demand for mental health services for: a. anxiety disorders and PTSD (267%) b. insomnia (356%) c. substance misuse (RR 1.44) d. suicidal behavior (RR not reported) after natural disasters for people > 60 years old.
Culqui et al. 2017 [65]	Madrid, Spain, 2001–2009	1,183 emergency admissions related to AD	Emergency admissions related to AD and exposure to PM_2.5_, PM_10_, NO_2_, O_3_ and daily temperature	Significant association of emergency AD-related admissions with PM_2.5_ levels > 25 µg/m^3^ at lag day 2 (RR 1.38) For an IQR of 1.33 ˚C above heatwave temperature of 34 ˚C, emergency AD-related admissions increased by 23.1% at lag day 3.	Age not reported; however, the study focused on patients with Alzheimer’s disease.
Dang et al., 2022 [61]	Ho Chi Minh City, Vietnam, 2017–2019	7,780 hospital admissions for mental and behavioral disorders	Effect of heatwaves occurrence (main effect) and duration (added effect) on mental health hospitalizations	All-cause mental health hospitalizations rose during heatwaves (RR 1.62) and slightly increased with longer heatwave duration (RR 1.08).	Individuals aged 18–60 were at higher risk for main effect, but older adults (> 60 years) had higher risk with longer heatwave duration (RR 1.61).
Gong et al. 2022 [13]	England, 1998–2009	Not reported	Risk of dementia-related hospitalization during heatwaves	The risk of dementia-related hospitalization increased 4.5% for every 1 ˚C above 17 ˚C.	Risk increased by 4.84% for adults aged 75–84 and by 4.83% for adults ≥ 85 years old vs. 2.27% for 16–74-year-olds.
Lee et al. 2018 [64]	Six cities in South Korea, 2003–2013	166,579 mental health admissions	Mental health emergency admissions and daily temperatures	14.6% of admissions were attributed to extremely hot temperatures (above the 99th percentile of the mean).	19.1% of emergency mental health admissions for adults > 65 years old were attributed to extremely hot temperatures
Liu et al. 2019 [62]	Jinan, China 2010	19,569 mental health admissions (3,573 of which occurred during 1 of 4 heatwaves)	Risk of hospital visits for mental illness during heatwaves, by demographic factors that included age	ORs ranged from 2.2 to 3.2 at lag day 2 and 3 for the heatwaves recorded.	Adults > 65 years old had a 3-times higher risk of admission compared to younger adults (OR 3.0).
Qiu et al. 2022 [63]	U.S., 2000–2016	165,572 urgent or emergency hospitalizations for schizophrenia; 458,492 admissions for depression; 166,833 admissions for bipolar disorder	Association of short-term exposure to air pollution (PM_2.5_, NO_2_, O_3_) and ambient temperature with acute hospitalizations for psychiatric disorders among U.S. Medicare beneficiaries	In the cold (but not warm) season: -For each 5 °C increase in short-term exposure to cold season temperature, admission RR increased by 3.66% for depression, 3.52% for bipolar disorder, and 3.03% for schizophrenia. -Significantly increased admission rates for depression, bipolar disorder, and schizophrenia with increased short-term exposure to PM_2.5_, and for depression and schizophrenia with elevated short-term NO_2_ exposure.	Only adults ≥ 65 years old were included
Schmeltz and Gamble 2017 [16]	U.S., 2001–2010	770 hospitalizations for concurrent mental illness and heat-related illness	Hospitalization for mental illness and co-occurring heat-related illness	Increased frequency of hospitalization for people with dementia, schizophrenia, and substance misuse co-occurring with heat-related illness.	RR 2.94 for 65–74-year-olds; RR 2.58 for adults ≥ 75 years old; RR 1.18 for adults ≥ 75 years old with psychosis^b^, all compared to 18–39-year-olds.
Wei et al. 2019 [66]	New England, U.S 2001–2011	3,069,816 Medicare enrollees	Effects of seasonal mean temperature and temperature variability on dementia-related admissions among U.S. Medicare beneficiaries	Lower risk of dementia-related hospitalizations with higher-than-average temperature; higher risk of hospitalization in zip codes with higher temperature variation.	Only adults ≥ 65 years old were included.
Xu et al. 2019 [69]	Brisbane, Australia, 2005–2013	907 hospitalizations of patients with AD	Effects of heatwaves (temperature higher than 95th percentile ≥ 2 days) on hospitalizations and post-discharge deaths	Hospitalizations increased 51% (2%-126%). Post-discharge deaths increased 269% (76% - 665%). Risks were further increased by higher heatwave temperature and living in areas with little vegetation. Death rates were higher in women than men.	93% of the hospitalized patients and 98.4% of those who died were ≥ 65 years old. All the patients who died were ≥ 55 years old.

*AD* Alzheimer’s disease, *IQR* interquartile range, *NO*_*2*_ nitrogen dioxide, *O*_*3*_ ozone, *OR* odds ratio, *PM*_*2.5*_ particulate matter with diameter smaller than 2.5 µm, *PM*_*10*_ particulate matter with diameter smaller than 10 µm, *PTSD* posttraumatic stress disorder, *RR* relative risk, *SO*_*2*_ sulphur dioxide

^a^Lag = the time in days after exposure to a variable, such as a heatwave, has ended

^b^Includes dementia and primary psychotic disorders

Extreme heat and air pollution have also been associated with heightened risk for suicide. A systematic review and meta-analysis that included studies with all age groups revealed that each 1% increase in temperature was significantly associated with a 1% increase in suicide rates [25]. This review also found a nonsignificant association between sunlight duration and suicides [25]. High ambient temperatures were associated with suicide deaths among adults ≥ 65 years old in Hong Kong [70]. And in a recent analysis of 28,670 suicide deaths in Korea, increased exposure to PM_2.5_ was significantly associated with suicide deaths, particularly among those 65 years old or older [71]. Excessive heat is also associated with a rise in violent crimes, including homicide, which can target older adults as well [72].

Given these findings, educating communities, in particular individuals with major NCDs and SMI and their caregivers regarding the health impacts of climate change and developing individualized emergency plans may reduce the risk of hospitalization and adverse medical or psychiatric outcomes. Additionally, health systems could better prepare for the rising demand in services [18].

In addition to the increased likelihood of being hospitalized for a mental health concern, meteorological conditions may increase risk for aggression and use of coercive measures such as physical restraints on inpatient psychiatric units. One study explored the number of aggressive incidents that occurred in six psychiatric hospitals in Germany totaling 1,007 beds, from 2007 to 2019. The authors uncovered significantly more aggressive incidents on days with temperatures ≥ 30 °C (86 F), compared to days with lower temperatures [73]. A study conducted in Poland also found lower atmospheric pressure and foehn (warm, dry) winds to be associated with increased use of restraints on an inpatient psychiatric unit [74]. Neither study presented age-stratified data.

## Outpatient Settings

Older adults may present in outpatient settings with various psychiatric symptoms related to climate change, although they may be more likely to seek primary care, rather than specialty services. Common climate-related mental health manifestations include anxiety, depression, acute or posttraumatic stress symptoms, and dysregulated sleep. Psychotic features can occur, albeit less often. Older individuals are less likely than younger generations to report climate anxiety (anxiety that occurs in reaction to anthropogenic climate change). Of note, climate anxiety is not considered a psychiatric diagnosis currently [2].

Table 3 illustrates the contribution of different climate-related factors to psychiatric presentations in older adults, based on current evidence [7–10, 12, 15, 19–24, 42, 43, 51, 75–87].

**Table 3:** Common psychiatric manifestations associated with climate change seen in older adults in outpatient settings

**Psychiatric manifestations**	**Extreme temperatures**	**Hurricanes and floods**	**Wildfires**	**Drought**	**Air pollution**
Adjustment disorders [19]		X	X		
Anxiety symptoms [21, 24, 75–77]	X	X	X	X	
Depressive symptoms [10, 20, 22, 77–79]	X	X	X	X	X
Posttraumatic stress symptoms [19–22, 75, 76, 78, 80]	X	X	X		
Psychotic symptoms [24, 51]		X	X		
Sleep disturbances^a^ [20, 76, 80–82]	X	X	X	X	X
Substance use [12, 75, 78, 83]	X	X	X	X	
Suicidality [12, 22, 24, 75, 77]	X	X		X	
Cognitive impairment [9, 15, 42, 43, 84–87]	X	X^b^	X		X

^a^Includes sleep onset, maintenance, duration, and quality, REM activity, and disordered breathing

^b^After displacement and relocation

The cognitive impacts of climate change are less known, but increasingly studied. Exposure to acute or chronic ambient air pollution has been linked to neuroinflammation, accelerated atherosclerosis, thrombosis activation, increased oxidative stress, and neurodegenerative processes [8, 9, 40]. Air pollution has been implicated in up to one third of the stroke burden and one fifth of dementia cases worldwide [7]. People aged 65–79 and those with prior strokes living in low- to middle-income countries are believed to be most vulnerable to the cerebrovascular effects of air pollution [7]. Large epidemiological studies have uncovered the association of various air pollutants with cognitive deficits in middle-aged and older adults, as well as with cognitive decline over time [42, 43]. A recent systematic review and meta-analysis revealed significant associations between chronic elevated PM_2.5_ exposure and all-cause dementia, AD, and Parkinson’s disease (PD) incidence, in addition to a significant association of PM_10_ exposure with vascular dementia rates [9]. A large U.S. cohort study which followed adults ≥ 50 years old without dementia from 1998 to 2016 found that higher residential PM_2.5_ levels, particularly from wildfires and agriculture sources, were associated with incident dementia [85]. This was the first investigation of source-specific PM_2.5_ exposure risk and cognitive impairment. Moreover, a study of cognitively intact adults ≥ 60 years old in the Los Angeles area showed negative associations of PM_2.5_, NO_2,_ and O_3_ levels with performance in several cognitive domains [86]. Heatwaves can increase risk of cognitive impairment among older adults as well [87].

Aging individuals who survive natural disasters may also present with impaired cognition, as noted in our vignette. Anxiety, depression, and poor sleep quality all affect cognitive functioning. Relocation and social isolation contribute to new onset or exacerbation of preexisting cognitive deficits [84], while people with major NCDs may exhibit more severe behavioral disturbances [88]. A brief screening test that is independent of education level, such as the Mini-Cog [89] can help triage individuals with unknown history. Establishing the cognitive baseline will be difficult in the absence of family informants or medical records, especially for older adults who present with delirium or disorganized thinking. In the office or inpatient units, the MMSE or Montreal Cognitive Assessment (MoCA) can be helpful [90]. The Saint Louis University Mental Status (SLUMS) is a newer, education-independent test which has also been studied with minoritized populations [91]. Screening tests may not provide an accurate representation of the patients’ cognitive status, given the potential confounding factors discussed above, and should be followed by neuropsychological evaluation, if deemed appropriate.

### Management

Studies that focus on interventions for older adults affected by climate change are scarce. More research is needed to fill this gap. Some directions for further study can be found in existing publications. Feder and colleagues [92] provided a blueprint for addressing climate anxiety, which includes normalizing affect, cognitive restructuring, and discussing the “butterfly effect” (i.e., small actions taken in the present may have a large effect in the future in complex systems). Clinicians can incorporate discussions about climate change effects in their routine practice and educate patients regarding the increased risk of heat stroke or complications with psychotropic medications [93] (also see Table 1). Health care professionals can help older patients develop emergency plans, locate shelters and cooling stations in advance, and be prepared to evacuate in case of extreme weather events. Several simple methods for regulating temperature overnight during heatwaves, such as taking a cool or lukewarm shower before bed, hydrating during the day, and directing fans over bare skin can reduce adverse outcomes [94].

There are few psychosocial interventions designed to support natural disaster survivors. CBT has been best studied and shown to be beneficial in reducing anxiety, depressive, and PTSD symptoms, including for older adults and people with SMI [17, 95–98]. Hamblen and colleagues developed a modified approach called CBT for post-disaster distress (CBT-PD) after Hurricane Katrina [95]. This intervention is delivered over 8–12 sessions and focuses on identifying and counteracting maladaptive beliefs related to the disaster. CBT-PD has also been used in the aftermath of Hurricane Sandy, with 40% of participants being ≥ 60 years old [96]. CBT-PD was effective in reducing moderate-to-severe PTSD symptoms, and improvements were maintained at 2 years [96]. After devastating wildfires in Fort McMurray, Alberta, Canada, an online CBT program called RESILIENT was also successful in decreasing PTSD, depression, insomnia, and anxiety symptoms; however, mean participant age in this study was 45 years [99]. Older adults, including those with neurodegenerative diseases such as PD had high adoption rates of online therapeutic interventions during the COVID-19 pandemic [100], and online group CBT for PTSD has demonstrated comparable effectiveness to in-person delivery [101]. Other nonpharmacological approaches used with disaster survivors have included psychoeducation, eye movement desensitization and reprocessing, meditation and relaxation techniques, yoga, and supportive therapy coupled with behavioral activation [22, 98]. Additionally, prospective studies of elderly relocated after the Great East Japan Earthquake showed that walking ≥ 30 min/day and being engaged in out-of-home activities ≥ 3 days/week were protective against cognitive decline 2 years post-disaster [84].

Pharmacological management of the psychiatric symptoms discussed above is similar to non-climate change-related conditions, as there is very limited evidence to support specific recommendations. Selective serotonin reuptake inhibitors or serotonin-norepinephrine reuptake inhibitors are the first line for anxiety, depressive, and posttraumatic stress symptoms [102]. Prazosin can also help improve nightmares and other PTSD symptoms such as hyperarousal and sleep disturbances [103]. Substance misuse needs to be addressed, as it can have other physical or psychiatric deleterious effects (e.g., worsened sleep, cognition, mood, anxiety, impulsivity, psychosis, and increased suicide risk).

Older adults can encounter many barriers to accessing mental health care in the aftermath of disasters: self-reliance, stigma, shortage of services, lack of transportation, financial pressures, and lack of information about resources [17, 20, 27]. Establishing mental health emergency points in hard-hit areas and using telehealth and other remote service modalities can improve access [17]. Other potential strategies include educating health care providers, embedding mental health clinicians in primary care settings, increasing mental health awareness, and broadly sharing information about resources [20]. Peer support programs have also been helpful after human-caused or weather-related disasters [17, 97]. Federal emergency programs and expanding insurance coverage for people living in affected areas are also crucial for recovery [17].

Although climate change brings numerous challenges and threats, older adults are very resilient, perhaps more so than younger people [104, 105]. A recent study conducted in a sample of U.S. Gulf Coast residents (mean age 58.4 ± 16, range 18–96) surveyed after two successive hurricane seasons found that older age was associated with significantly fewer self-reported PTSD and depressive symptoms post-disaster, and the latter association was stronger among those who had encountered hurricane-related adversities than those who had not [105]. Social support and social cohesion have been shown to buffer the mental health impacts of natural disasters among aging adults [21, 106, 107]. Neighborhood green spaces can help improve well-being, enhance resilience, and protect older adults against developing post-disaster PTSD [108, 109].

Finally, older individuals are engaged in their communities and volunteer activities. The project Retirees in Service to the Environment (RISE) provided environmental awareness workshops to 149 older individuals at eleven sites in New York State and Florida, leading to an increase in the number of hours volunteered and multiple successful community stewardship projects [110].

## Conclusions

A growing body of research regarding climate change-related clinical presentations in older adults exists, underscoring the increased risk for medical and psychiatric complications. The extant body of work focuses on extreme temperatures (including heatwaves), weather-related disasters, and air pollution. Anxiety, depressive symptoms, sleep disturbances, posttraumatic, and cognitive deficits are the most common symptoms associated with climate change. Older adults with major neurocognitive disorders and those with preexisting psychiatric illness have a higher risk of psychiatric hospitalizations after exposure to high temperatures and air pollution. Educating clinicians, patients, and caregivers is paramount, while keeping in mind that aging adults may have varying risk perceptions regarding climate change. Few evidence-based management strategies (mainly psychosocial interventions) exist, and there are no medication randomized controlled trials. Further work is needed to develop culturally appropriate interventions for older disaster survivors, as well as strategies to enhance individual and collective resilience. Many opportunities to research and improve the care of older adults in this constantly changing world remain.

## Data Availability

No datasets were generated or analysed during the current study.
